# MXene‐Integrated Perylene Anode with Ultra‐Stable and Fast Ammonium‐Ion Storage for Aqueous Micro Batteries

**DOI:** 10.1002/advs.202305524

**Published:** 2023-11-14

**Authors:** Ke Niu, Junjie Shi, Long Zhang, Yang Yue, Shuyi Mo, Shaofei Li, Wenbiao Li, Li Wen, Yixin Hou, Li Sun, Shuwen Yan, Fei Long, Yihua Gao

**Affiliations:** ^1^ College of Materials Science and Engineering Guangxi Key Laboratory of Optical and Electronic Materials and Devices and Collaborative Innovation Center for Exploration of Nonferrous Metal Deposits and Efficient Utilization of Resources Guilin University of Technology Guilin 541004 China; ^2^ School of Physics and Center for Nanoscale Characterization & Devices (CNCD) Wuhan National Laboratory for Optoelectronics (WNLO) Huazhong University of Science and Technology (HUST) Wuhan 430074 China; ^3^ Information Materials and Intelligent Sensing Laboratory of Anhui Province Key Laboratory of Structure and Functional Regulation of Hybrid Materials of Ministry of Education Institutes of Physical Science and Information Technology Anhui University Hefei 230601 China

**Keywords:** aqueous micro batteries, flexibility, PTCDA/Ti_3_C_2_T_x_ MXene, ultra‐stable and fast NH_4_^+^ storage

## Abstract

The aqueous micro batteries (AMBs) are expected to be one of the most promising micro energy storage devices for its safe operation and cost‐effectiveness. However, the performance of the AMBs is not satisfactory, which is attributed to strong interaction between metal ions and the electrode materials. Here, the first AMBs are developed with NH_4_^+^ as charge carrier. More importantly, to solve the low conductivity and the dissolution during the NH_4_^+^ intercalation/extraction problem of perylene material represented by perylene‐3,4,9,10‐tetracarboxylic dianhydride (PTCDA), the Ti_3_C_2_T_x_ MXene with high conductivity and polar surface terminals is introduced as a conductive skeleton (PTCDA/Ti_3_C_2_T_x_ MXene). Benefitting from this, the PTCDA/Ti_3_C_2_T_x_ MXene electrodes exhibit ultra‐high cycle life and rate capability (74.31% after 10 000 galvanostatic chargedischarge (GCD) cycles, and 91.67 mAh g^−1^ at 15.0 A g^−1^, i.e., capacity retention of 45.2% for a 30‐fold increase in current density). More significantly, the AMBs with NH_4_^+^ as charge carrier and PTCDA/Ti_3_C_2_T_x_ MXene anode provide excellent energy density and power density, cycle life, and flexibility. This work will provide strategy for the development of NH_4_^+^ storage materials and the design of AMBs.

## Graphical abstract

A new type of MXene‐integrated perylene anode (PTCDA/Ti_3_C_2_T_x_ MXene) is developed that indicates ultra‐stable and fast NH_4_^+^ storage. The PTCDA/Ti_3_C_2_T_x_ MXene anode provides an entirely new method to solve the low conductivity and the dissolution of perylene anode, which also provides an effective direction for building excellent aqueous NH_4_^+^ micro batteries.

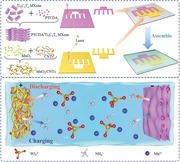

## 1 Introduction

Rapid development of smart wearable and integrated electronics requires energy storage devices (ESDs) to be more safe and miniaturized.^[^ ^1^, ^2^, ^3^, ^4^ ^]^ Aqueous micro batteries (AMBs) inheriting the advantages such as excellent safety, cost‐effectiveness, and power density of aqueous batteries are promising ESDs.^[^ ^5^, ^6^ ^]^ Unfortunately, the development of AMBs is still relatively backward, so the research and development of AMBs was required to pay more attention.^[^ ^7^, ^8^, ^9^, ^10^ ^]^ As well known, the performance of ESDs is always attributed to the interaction between the current carrier and the electrode material, so developing efficient charge carriers and electrode materials is a significant means.

So far, most AMBs or aqueous batteries use metal ions such as Li^+^, Na^+^, K^+^, Zn^2+,^ and Al^3+^ as charge carriers.^[^ ^11^, ^12^, ^13^, ^14^, ^15^, ^16^ ^]^ However, the strong interaction between metal ions and the electrode materials limits their intercalation/extraction kinetics, which further limits their performance.^[^ ^17^, ^18^ ^]^ For the past few years, aqueous batteries using nonmetallic ions as charge carriers have been proposed to develop faster ESDs.^[^ ^19^, ^20^, ^21^ ^]^ Among possible nonmetallic ions, the NH_4_^+^ as a charge carrier is of particular interest due to their extremely abundant natural abundance, lower molar mass (18 g mol^—1^), and smaller hydrated ion radius (3.31 Å) than most metal ions which enhance adsorption reactions.^[^ ^22^ ^]^ Compared to alkaline or acidic electrolytes, aqueous NH_4_^+^ solutions are less corrosive and have a higher potential for oxygen evolution reaction (OER) or lower potential for hydrogen evolution reaction (HER), which widens the electrochemical stability window.^[^ ^23^ ^]^ Due to its tetrahedral structure, NH_4_^+^ results in a different intercalation chemistry of NH_4_^+^ in the main electrode material from conventional metal ion carriers. So, NH_4_^+^ is considered as a suitable carrier for AMBs with a rocking‐chair form. Among many NH_4_^+^ storage materials, organic materials represented by perylene with abundant resources and easy synthesis are considered to be the most promising anode materials. Wu and co‐workers first reported 3,4,9, 10‐Perylenetetracarboxylic diimide (PTCDI) as a NH_4_^+^ storage material in 2017.^[^ ^23^ ^]^ Then, alloxazine (ALO), polyaniline (PANI), and poly(1,4,5,8‐naphthalenetetracarboxylic anhydride naphthylamine) imine (PNNI) have been investigated as electrode materials for NH_4_^+^ storage.^[^ ^24^, ^25^, ^26^ ^]^ Although organic material has shown favorable NH_4_^+^ storage capacity, there are still many challenges, such as low conductivity and dissolution during the ion intercalation/extraction.^[^ ^27^ ^]^ Generally, the integration of organic molecules onto a conductive skeleton can effectively enhance electron transfer and guarantee a stable structure. As a new 2D material, Ti_3_C_2_T_x_ MXene has better electric conductivity (≈10^4^ S cm^−1^) and adsorption capacity than conventional carbon‐based conductors due to the presence of transition metals.^[^ ^28^, ^29^, ^30^, ^31^ ^]^ More importantly, the polar surface functional groups such as –F, –OH, etc. give Ti_3_C_2_T_x_ MXene better hydrophily, which is conducive to the contact between aqueous electrolyte and active substance.^[^ ^32^, ^33^, ^34^, ^35^, ^36^ ^]^ Therefore, Ti_3_C_2_T_x_ MXene is superior to conventional carbon‐based conductors in terms of hosting guest compounds and electrolyte wettability. However, it remains a challenge to integrate the organic phase perylene and aqueous phase Ti_3_C_2_T_x_ MXene to build ultra‐stable and fast anode. Although MXene‐integrated perylene anode with ultra‐stable and fast NH_4_^+^ storage for AMBs have been developed as described above.

Here, we develop MXene‐integrated perylene anode for AMBs, which is assembled by Ti_3_C_2_T_x_ MXene and perylene‐3,4,9,10‐tetracarboxylic dianhydride (PTCDA). It is worth emphasizing that Ti_3_C_2_T_x_ MXene is dispersed in N, N‐Dimethylformamide (DMF) by solvent displacement (Ti_3_C_2_T_x_ MXene/DMF), which allows Ti_3_C_2_T_x_ MXene solution to have colloidal properties and retain hydrophilic functional groups. More significantly, Ti_3_C_2_T_x_ MXene can greatly improve the stability and conductivity of PTCDA, so that PTCDA/Ti_3_C_2_T_x_ MXene electrodes has ultra‐high cycle life (74.31% after 10 000 GCD cycles at the current density of 15.0 A g^−1^) and rate capability rate capability (91.67 mAh g^−1^ at 15.0 A g^−1^, i.e., capacity retention of 45.2% for a 30‐fold increase in current density), which is in agreement with the calculated results of density functional theory (DFT). In addition, ex situ X‐ray Photoelectron Spectroscopy (XPS) and scanning electron microscope (SEM) characterization demonstrate the reversible storage of NH_4_^+^. For cathode materials, the MnO_2_/CNTs (Nanjing XFNANO Materials) are selected because of their large capacity, high natural abundance, low cost, and low toxicity.^[^ ^37^ ^]^ Therefore, AMBs based on PTCDA/Ti_3_C_2_T_x_ MXene anode are manufactured by laser engraving process, providing excellent energy density (66.05 µWh cm^−2^) and power density (2.61 mW cm^−2^), cycle life (90.43% after 2500 GCD cycles at current density of 5.0 mA cm^−2^) and flexibility (94.79% under 180°). We believe that this work will provide a strategy for the development of NH_4_^+^ storage materials and the design of AMBs.

## 2 Results and Discussion

**Figure 1:**
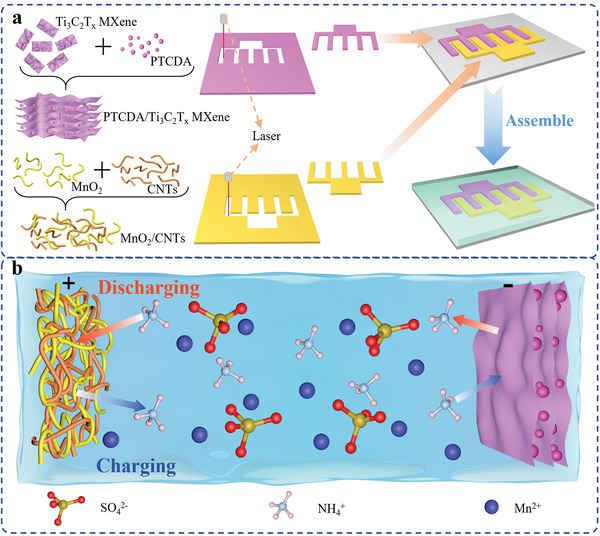
The manufacturing process, structure, and working principle of AMBs. a) The fabrication process of AMBs and b) The structure and working principle of AMBs.

The manufacturing processes of AMBs are shown in **Figure** **1**a. First, PTCDA/Ti_3_C_2_T_x_ MXene anode is prepared by mixing PTCDA and Ti_3_C_2_T_x_ MXene. Meanwhile, the MnO_2_/CNTs cathode is prepared by mixing MnO_2_ and CNTs. Then, the laser direct carving technology was used to achieve the finger‐like PTCDA/Ti_3_C_2_T_x_ MXene anode and MnO_2_/CNTs cathode. After transferring the finger‐like PTCDA/Ti_3_C_2_T_x_ MXene anode and MnO_2_/CNTs cathode to polyurethane film and then coating the polyacrylamide (PAM) hydrogel, an AMB was successfully obtained. The working principles of AMBs are shown in Figure 1b. At charging process, the NH_4_^+^ is extracted from the MnO_2_/CNTs cathode and intercalated into the PTCDA/Ti_3_C_2_T_x_ MXene anode. At discharging process, the NH_4_^+^ is extracted from the PTCDA/Ti_3_C_2_T_x_ MXene anode and intercalated into the MnO_2_/CNTs cathode. It is worth emphasizing that the addition of a small amount of manganese salt in the electrolyte can greatly improve the stability of the cathode, which can also improve the cycle life of AMBs.

**Figure 2:**
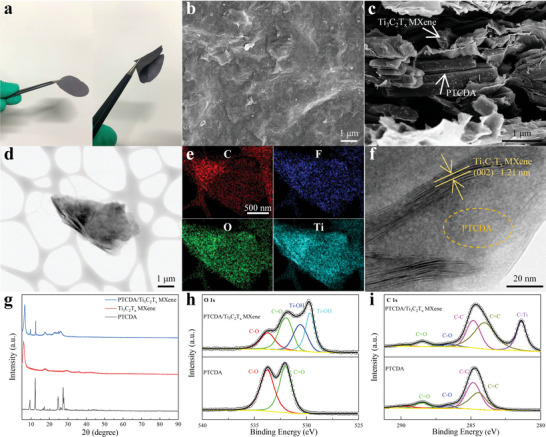
The characterization of PTCDA/Ti_3_C_2_T_x_ MXene free‐standing films. a) The optical photographs, the SEM images of b) surface and c) cross section, d)TEM images, e) STEM‐mapping images, f) HRTEM images of PTCDA/Ti_3_C_2_T_x_ MXene. g) XRD pattern, XPS spectra of h) O 1s and i) C 1s of PTCDA/Ti_3_C_2_T_x_ MXene and Ti_3_C_2_T_x_ MXene free‐standing films.

The successful preparation of PTCDA/Ti_3_C_2_T_x_ MXene anode with ultra‐stable and fast NH_4_^+^ storage is particularly important for AMBs. So, aqueous Ti_3_C_2_T_x_ MXene was synthesized by the common HCl/LiF etching method (Figures S1 and S2, Supporting Information). Then Ti_3_C_2_T_x_ MXene/DMF was synthesized by solvent substitution.^[^ ^38^ ^]^ Ti_3_C_2_T_x_ MXene/DMF has a Dendar effect similar to aqueous MXene, indicating that Ti_3_C_2_T_x_ MXene is fully and uniformly dispersed in DMF (Figure S3). As shown in Figure S4 (Supporting Information), the transmission electron microscope (TEM) images of commercialized PTCDA exhibit a classical amorphous structure. The PTCDA/Ti_3_C_2_T_x_ MXene free‐standing films were further prepared by simple solution mixing and extraction filtration. As shown in **Figure** **2**a, the optical photographs of PTCDA/Ti_3_C_2_T_x_ MXene free‐standing films show that it has excellent flexibility. As shown in Figure 2b, the uniform surface of free‐standing PTCDA/Ti_3_C_2_T_x_ MXene film in the SEM images indicates that PTCDA and Ti_3_C_2_T_x_ MXene are evenly mixed. The SEM image of the cross section in Figure 2c indicates free‐standing PTCDA/Ti_3_C_2_T_x_ MXene films are a uniform stack of PTCDA and Ti_3_C_2_T_x_ MXene. Subsequently, free‐standing PTCDA/Ti_3_C_2_T_x_ MXene films were further characterized by TEM (Figure 2df). The uniform distribution of C, O, and Ti in STEM‐mapping (Figure 2e) further demonstrates the loading of PTCDA on the Ti_3_C_2_T_x_ MXene, which is also verified by the HRTEM image (Figure 2f). The structure changes of PTCDA and PTCDA/Ti_3_C_2_T_x_ MXene were analyzed by X‐ray diffraction (XRD) pattern (Figure 2g). The XRD pattern of PTCDA/Ti_3_C_2_T_x_ MXene shows characteristic peaks of both PTCDA and Ti_3_C_2_T_x_ MXene, indicating the successful combination.^[^ ^39^, ^40^, ^41^ ^]^ Accordingly, the XPS full spectrum in Figure S5 (Supporting Information) shows that the PTCDA/Ti_3_C_2_T_x_ film displays typical peaks of PTCDA and Ti_3_C_2_T_x_. As shown in Figure 2h, in the XPS spectra of O 1s, the PTCDA exhibits C─O─C and C═O groups.^[^ ^42^ ^]^ And PTCDA/Ti_3_C_2_T_x_ MXene exhibits not only these peaks, but also Ti─OH and Ti═O groups, indicating that the successful integration of PTCDA and Ti_3_C_2_T_x_ MXene. In addition, the C‐Ti group can be seen in the XPS spectra of C 1s in PTCDA/Ti_3_C_2_T_x_ MXene in Figure 2i, which also indicates the successful integration. The FTIR spectrum of PTCDA and PTCDA/Ti_3_C_2_T_x_ MXene in Figure S6 (Supporting Information) also accounts for their successful integration. The performances of free‐standing PTCDA/Ti_3_C_2_T_x_ MXene films were further tested to demonstrate the superiority of MXene as a conductive framework. As shown in Figure S7 (Supporting Information), the contact Angle between free‐standing PTCDA/Ti_3_C_2_T_x_ MXene film with the electrolyte was tested, indicating the advantages of Ti_3_C_2_T_x_ MXene in terms of hosting guest compounds and electrolyte wettability.

**Figure 3:**
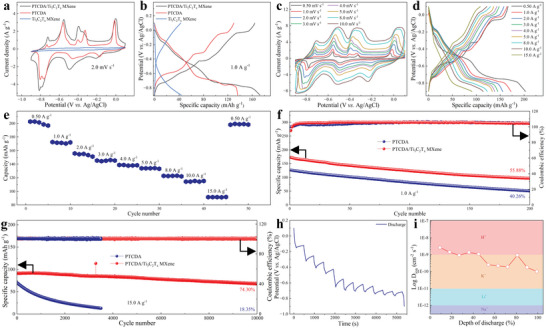
The electrochemical performance of PTCDA/Ti_3_C_2_T_x_ MXene. The a) CV curves at 2.0 mV s^−1^ and b) GCD curves at 1.0 A g^−1^ of Ti_3_C_2_T_x_ MXene, PTCDA and PTCDA/Ti_3_C_2_T_x_ MXene. c) CV curves from 0.50 mV s^−1^ to 10.0 mV s^−1^, d) GCD curves from 0.50 A g^−1^ to 15.0 A g^−1^, e) rate performance, f) cycle life at 1.0 A g^−1^ and g) 15.0 A g^−1^ of PTCDA/Ti_3_C_2_T_x_ MXene. h) GITT and i) corresponding calculated NH_4_^+^ diffusion coefficient during discharging and the comparison with other ions.

The electrochemical performances of Ti_3_C_2_T_x_ MXene, PTCDA and PTCDA/Ti_3_C_2_T_x_ MXene were further tested. As shown in **Figure** **3**a,b, the cyclic voltammetry (CV) curves at 2.0 mV s^−1^ and GCD curves at 1.0 A g^−1^ of PTCDA/Ti_3_C_2_T_x_ MXene is greater than that of single MXene and single PTCDA. In addition, the electrochemical performances of different components of PTCDA/Ti_3_C_2_T_x_ MXene have also been studied. As shown in Figure S8 (Supporting Information), when the ratio of PTCDA to Ti_3_C_2_T_x_ MXene is 6.0:4.0, the electrochemical performance of PTCDA/Ti_3_C_2_T_x_ MXene is the best. If there is no special emphasis, PTCDA/Ti_3_C_2_T_x_ MXene will use the optimal ratio in the rest of the discussion. The CV curves from 0.50 to 10.0 mV s^−1^ and GCD curves from 0.50 to 15.0 A g^−1^ of PTCDA/Ti_3_C_2_T_x_ MXene free‐standing film were tested. As shown in Figure 3c, all CV curves have similar shapes and increase area with scan rate. As shown in Figure 3d, the GCD curves of PTCDA/Ti_3_C_2_T_x_ MXene free‐standing film showed two low potential discharge platforms, indicating the feasibility of PTCDA/Ti_3_C_2_T_x_ MXene free‐standing film as anode. The rate capability of PTCDA/Ti_3_C_2_T_x_ MXene free‐standing film was further tested in Figure 3e. When the current density increased from 0.50 to 15.0 A g^−1^, the specific capacity decreased from 202.79 to 91.67 mAh g^−1^, indicating that the excellent capacity and rate capability of free‐standing PTCDA/Ti_3_C_2_T_x_ MXene films. After several cycles and then moving back to 0.50 A g^−1^, the specific capacity was recovered to 199.01 mAh g^−1^ and the recovery rate was 98.14%, which exhibited that PTCDA/Ti_3_C_2_T_x_ MXene free‐standing films the has superior rate capability. As shown in Figure 3f, the stability of PTCDA and free‐standing PTCDA/Ti_3_C_2_T_x_ MXene films are tested at a current density of 1.0 A g^−1^, indicating the excellent cycle life of free‐standing PTCDA/Ti_3_C_2_T_x_ MXene films. As shown in Figure 3g, to further demonstrate the excellent stability of free‐standing PTCDA/Ti_3_C_2_T_x_ MXene films, the cycle life was tested at a higher current density of 15.0 A g^−1^. After 10 000 GCD cycles, the capacity retention of the free‐standing PTCDA/Ti_3_C_2_T_x_ MXene films remains at 74.30%, which is better than PTCDA (18.35% after 3500 cycles). Meanwhile, the Ti_3_C_2_T_x_ Mxene films also exhibt excellent stability, indicating that Ti_3_C_2_T_x_ can effectively enhance electron transfer and guarantee a stable structure. Figure S10 (Supporting Information) exhibits the SEM images of the PTCDA/Ti_3_C_2_T_x_ MXene after cycling at different current densities. The PTCDA/Ti_3_C_2_T_x_ MXene after 15.0 A g^−1^ cycling (1000 cycles) maintained a similar morphology to the original sample. But, the PTCDA in PTCDA/Ti_3_C_2_T_x_ MXene after 1.0 A g^−1^ cycling (200 cycles) had dissolved into small particles, which leads to capacity decay. In summary, higher charging and discharging depth is more likely to cause damage to the structure of PTCDA/Ti_3_C_2_T_x_ MXene. However, it is worth emphasizing that, using MXene to improve the stability and conductivity of PTCDA is an effective way. The Nyquist plot of free‐standing PTCDA/Ti_3_C_2_T_x_ MXene films is shown in Figure S11 (Supporting Information), indicating excellent electrical conductivity. The ion diffusion coefficient was investigated by galvanostatic intermittent titration technique (GITT) to reveal reaction kinetics (Figure 3h and Figure S12, Supporting Information). The calculated ion diffusion coefficient of NH_4_^+^ ion is as high as 10^−9^ to 10^−10^ cm^2^ s^−1^ (Figure 3i).^[^ ^43^, ^44^, ^45^, ^46^ ^]^ Compared with other monovalent metal charge carriers, NH_4_^+^ exhibits superior diffusion kinetics, which is the reason for its excellent rate capability.

**Figure 4:**
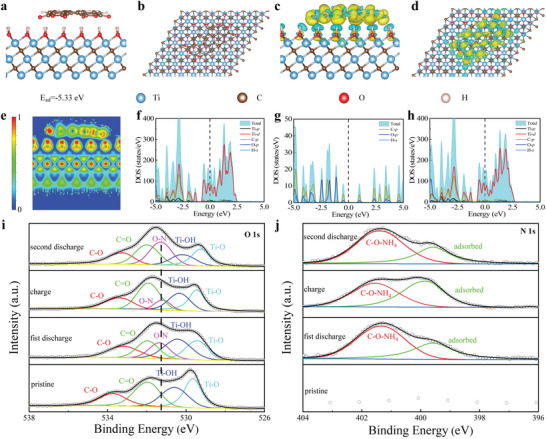
Optimized side‐viewed and top‐viewed (a,b) configuration with corresponding adsorption energy (c,d) charge density difference with the isosurface value of 6.63 × 10^−4^ e Å^−3^ of Ti_3_C_2_OH‐PTCDA composite, e) ELF of Ti_3_C_2_OH‐PTCDA in (010) plane under zero external field and DOS of f) Ti_3_C_2_OH, g) PTCDA, and h) Ti_3_C_2_OH‐PTCDA. i) The high‐resolution XPS spectra i) O 1s and j) N 1s of PTCDA/Ti_3_C_2_T_x_ during pristine, first discharge, charge, and second discharge.

The mechanism for PTCDA/Ti_3_C_2_T_x_ MXene free‐standing films with ultra‐stable and fast NH_4_^+^ storage is revealed by DFT calculation and XPS spectrum at different charged states. **Figure** **4**a,b displays the optimized crystal structure of Ti_3_C_2_OH‐PTCDA, where the O atoms of PTCDA are close to the H atoms of Ti_3_C_2_OH, the atomic plane of PTCDA is parallel to the surface of Ti_3_C_2_OH, the effective interactions between them cause the atoms of PTCDA to be in positions near the pristine plane and slightly distort ─OH functional groups of Ti_3_C_2_OH. The corresponding adsorption energy is −5.33 eV, which indicates its stable adsorption. The charge transfer occurs at the interface, where the H atoms mainly lose electrons while the C and O atoms gain them (Figure 4c,d). The ionic characters of Ti_3_C_2_OH and the covalent characters of PTCDA are obvious from the electron localization function (ELF) in Figure 4e, where the electron localization is visible and consistent with the charge transfer. Figure 4f–h displays the total and main projected density of states (DOS) to further explore the electronic structures. The DOS of Ti_3_C_2_OH indicates the electrical conductivity while PTCDA shows a bandgap of 0.86 eV. The DOS of Ti_3_C_2_OH‐PTCDA implies the conductivity of the composite, the Ti‐*d* orbitals make the main contribution around the Fermi level. One can see the atomic orbitals interactions of C‐*p*, O‐*p*, Ti‐*p*, and Ti‐*d* below the Fermi level. The binding energy, charge density difference, ELF, and DOS collectively reveal the strong interactions between Ti_3_C_2_OH and PTCDA. To elucidate the storage mechanism of NH_4_^+^ in the free‐standing PTCDA/Ti_3_C_2_T_x_ MXene films, the structural evolution was studied by the ex‐situ XPS characterization. Figure 4i,j shows the high‐resolution XPS spectra of PTCDA/Ti_3_C_2_T_x_ during pristine, fist discharge, charge, and second discharge. As shown in Figure 4i, during the discharging process, NH_4_^+^ and C═O generate O─N bonds. During the charge process, O─N bonds decrease, and then increase again during the second discharging process. In the image of XPS N 1s, the C‐O‐NH_4_ peak at 401.5 eV also changes reversibly with the charge–discharge process. This suggests that the NH_4_^+^ in the process of energy storage materials is reversible.

**Figure 5:**
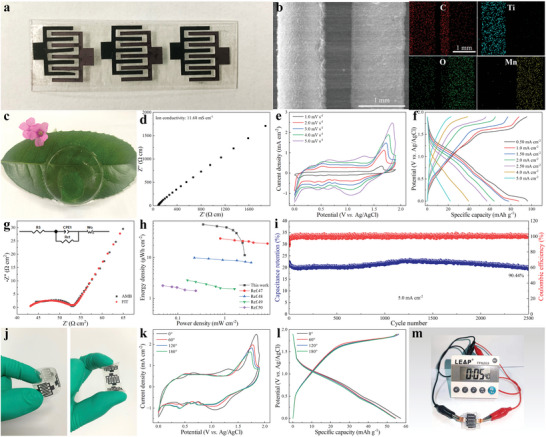
The characterization, electrochemical performance, and practicability of AMBs (a) photograph and b) SEM image and mapping images of the finger‐like electrodes. c) Optical photograph and d) EIS curves of PAM hydrogel. e) CV curves from 1.0 to 5.0 mV s^−1^, f) GCD curves from 0.50 to 5.0 mA cm^−2^, g) the Nyquist plot and equivalent circuits, h) energy and power density plot, and i) cycle life and coulomb efficiency of AMBs. j) photograph, k) CV curves, and l) GCD curves of AMBs under bending. m) The single AMB powers for counters.

In order to assemble AMBs with excellent performance, MnO_2_ and MnO_2_/CNTs were successfully prepared in Figures S13–S15 (Supporting Information). As shown in Figures S16 and S17 (Supporting Information), MnO_2_/CNTs cathodes also show excellent contact angles and electrochemical performance. More importantly, Figure S18 (Supporting Information) shows the XPS spectrum of MnO_2_/CNTs electrode at the charging and discharging states, indicating the energy storage form of MnO_2_/CNTs electrodes is the insertion/extraction of NH_4_^+^. Subsequently, charge matching was performed on PTCDA/Ti_3_C_2_T_x_ MXene anode and MnO_2_/CNTs cathode in aqueous electrolyte solution. Since the specific capacity of MnO_2_/CNTs cathode is smaller than that of PTCDA/Ti_3_C_2_T_x_ MXene anode, the mass ratio of PTCDA/Ti_3_C_2_T_x_ MXene anode to MnO_2_/CNTs cathode is set at 0.80 mg:2.80 mg according to the formula *m*_+_/*m*_‐_ = *C*_‐_Δ*V*_‐_/*C*_+_Δ*V*_+_. As shown in Figure S19 (Supporting Information), the CV curves of the anode and cathode at a scan rate of 2.0 mV s^−1^ exhibit that the changes of the anode and cathode are well balanced. As a proof of concept, aqueous batteries were assembled and tested in aqueous electrolyte solution. Figure S20a,b (Supporting Information) shows the CV curves at scan rates at 2.0 mV s^−1^ and the current densities at 1 .0 A g^−1^of the aqueous batteries that exhibit large peak current densities and good electrochemical performance (166.74 mAh g^−1^). Figure S20c (Supporting Information) shows aqueous batteries have very low resistance. The AMBs are fabricated using laser engraving free‐standing PTCDA/Ti_3_C_2_T_x_ MXene films and free‐standing MnO_2_/CNTs films and assembled with PAM gel electrolyte. As shown in **Figure** **5**a, the photograph of the finger‐like electrodes exhibits the microsize of AMBs. The SEM images and corresponding element map images of the finger‐like electrodes reveal that the PTCDA/Ti_3_C_2_T_x_ MXene anode and MnO_2_/CNTs cathode are uniformly and alternately integrated (Figure 5b). The PAM hydrogel electrolyte in Figure 5c is prepared by simply pre‐saturating the PAM film (Figure 5c). The EIS curves demonstrate that the PAM hydrogel electrolyte possesses a decent ionic conductivity of 11.68 mS cm^−1^. The CV curves from 1.0 to 5.0 mV s^−1^ and GCD curves from 0.50 to 5.00 A g^−1^ of AMBs were tested. As shown in Figure 5e, all CV curves have similar shapes and increase area with scan rate. The specific capacities of AMBs in Figure 5f are 96.11, 87.69, 77.98, 64.27, 57.20, 39.52, and 22.05 mAh cm^−2^ at current density 0.50, 1.0,1.5, 2.0, 2.5, 4.0, and 5.0 mA cm^−2^, respectively. The Nyquist plot and equivalent circuits of AMBs are shown in Figure 5g and its inset, indicating that the equivalent series resistance (Rs) and charge transfer resistance (Rct) were determined to be 43.13 and 9.91 Ω cm^2^, respectively. As shown in Figure 5h, the energy density of the AMBs can reach up to 66.05 µWh cm^−2^ at 0.344 mW cm^−2^ and 11.50 µWh cm^−2^ at 2.61 mW cm^−2^, which is much larger than similar MESDs.^[^ ^47^, ^48^, ^49^, ^50^ ^]^ To verify the stability of AMBs, the GCD cycles were performed at a current density of 10.0 mA cm^−2^. As shown in Figure 5i, after 2500 GCD cycles, the initial capacitance of AMBs still retain its 90.43%. Considering that ESDs often need to be operated in extreme conditions, single AMB was tested under bent. The optical photographs of the bend of AMB are provided in Figure 5j. In different bending states, the capacity of AMB changes very little, indicating that the battery has good flexibility (Figure 5k,l). Finally, to demonstrate the utility of AMBs, the single AMB was used to drive counters (Figure 5m).

## 3 Conclusions

In summary, we develop MXene‐integrated perylene electrode based on PTCDA and the Ti_3_C_2_T_x_ MXene/DMF. The introduction of Ti_3_C_2_T_x_ MXene/DMF with high conductivity and hydrophilicity was introduced as a conductive skeleton can greatly improve the stability and conductivity of PTCDA, so that PTCDA/Ti_3_C_2_T_x_ MXene electrodes has ultra‐high cycle life (74.31% after 10 000 GCD cycles at the current density of 15 A g^−1^) and rate capability (91.67 mAh g^−1^ at 15.0 A g^−1^, i.e., capacity retention of 45.2% for a 30‐fold increase in current density). Subsequently, DFT calculations and infiltration tests were used to support the above views. More importantly, ex situ XPS and SEM characterization was used to demonstrate the reversible storage of NH_4_^+^. Therefore, AMBs based on PTCDA/Ti_3_C_2_T_x_ MXene anode are manufactured by laser engraving process, providing excellent energy density (66.05 µWh cm^−2^) and power density (2.61 mW cm^−2^), cycle life (90.43% after 2500 GCD cycles at current density of 5.0 mA cm^−2^) and flexibility (94.79% uder 180°). This work will provide guidance for the development of NH_4_^+^ storage materials and the design of AMBs.

## Conflict of Interest

The authors declare no conflict of interest.

## Supporting information

Supporting Information

## Data Availability

The data that support the findings of this study are available from the corresponding author upon reasonable request.
