# Supplementary material for: MXene‐Integrated Perylene Anode with Ultra‐Stable and Fast Ammonium‐Ion Storage for Aqueous Micro Batteries
Source: Adv Sci (Weinh). 2023 Nov 14;11(1):2305524. doi: 10.1002/advs.202305524 (PMC10767440; doi:10.1002/advs.202305524)
Supplement: Supplementary file 1 — Supporting Information [file ADVS-11-2305524-s001.pdf]

ADVANCED SCIENCE Open Access

Supporting Information

for *Adv. Sci.*, DOI 10.1002/advs.202305524

MXene-Integrated Perylene Anode with Ultra-Stable and Fast Ammonium-Ion Storage for Aqueous Micro Batteries

Ke Niu, Junjie Shi, Long Zhang, Yang Yue, Shuyi Mo, Shaofei Li, Wenbiao Li, Li Wen, Yixin Hou, Li Sun, Shuwen Yan, Fei Long and Yihua Gao**

MXene-Integrated Perylene Anode with Ultra-Stable and Fast Ammonium-Ion Storage for Aqueous Micro Battery

Ke Niu, Junjie Shi, Long Zhang, Yang Yue, Shuyi Mo, Shaofei Li, Wenbiao Li, Li Wen, Yixin Hou, Li Sun, Shuwen Yan, Fei Long, Yihua Gao**

Experiments Section

1. Reagents and materials

Ammonium sulfate ($(NH_4)_2SO_4$), manganese sulfate monohydrate ($MnSO_4{\cdot}H_2O$,), potassium permanganate ($KMnO_4$), sodium dodecyl sulfate (SDS), hydrochloric acid (HCl), N,N-Dimethylformamide (DMF), Sulfuric acid (H_2SO_4), lithium fluoride (LiF, 98.5%), Acrylamide (AM), Potassium persulfate ($K_2S_2O_8$), and N, N′-methylenebisacrylamide (MBA) were purchased from Sinopharm Chemical Reagent Co., Ltd. CNTs and Ti_3AlC_2 power were purchased from Nanjing XFNANO Materials Tech. Co., Ltd and 11 Technology Co., Ltd respectively.

2. Synthesis of aqueous $Ti_3C_2T_x$ MXene and organic phase $Ti_3C_2T_x$ MXene

According to literature reports, the aqueous $Ti_3C_2T_x$ MXene were prepared by etching aluminum from Ti_3AlC_2 in HCl and LiF mixed solution. To put it simply, 1.0 g LiF was gently added to 20.0 mL HCl (9.0 M) and kept magnetic stirring until completely dissolved. Then, 1.0 g Ti_3AlC_2 was added to the above solution stably, stirred at the temperature of 35 °C for 24.0 h. After complete reaction, the mixture was centrifuged at 3500 rpm for 5.0 min again and again until the pH value reached about

6. After centrifugation, the sediment was redispersed in deionized water and ultrasonic processed for 1.0 h under certain condition (Ar atmosphere and below 35℃). Last, the above solution was centrifuged at 3500 rpm for 30.0 min again to obtain the supernatant of aqueous $Ti_3C_2T_x$ MXene.

The organic phase $Ti_3C_2T_x$ MXene is prepared by solvent displacement. The aqueous $Ti_3C_2T_x$ MXene was centrifuged at 10000 rpm for 1.0 h. The centrifuge tube was filled with 30.0 mL of DMF organic solvent and vigorously agitated until the residue was entirely dispersed. The centrifugation at 1000 rpm for 30 min was used to collect the lower density liquid after being repeated three times. Finally, the 1.0 mg mL^{-1} of MXene/DMF solution was reserved for later use.

3. Synthesis of MnO_2 and MnO_2/CNTs films

In a typical synthesis process, 3.0 mmol $MnSO_4 \cdot H_2O$ and 2.0 mL H_2SO_4 (0.50 M) were added to 60.0 mL deionized water under vigorous stirring for 30 min and then 20 mL 0.10 M $KMnO_4$ aqueous solution was slowly added into the above solution. The mixture was stirred for 2.0 h, followed by sonication for 30.0 min. The mixture was transferred to a Teflon-lined stainless-steel autoclave and heated at 120 ℃ for 12.0 h. After cooling down to room temperature, the obtained material was gathered via centrifugation and washed three times with deionized water, and dried in freeze.

4.0 mg CNTs, 6.0 mg MnO_2 nanowires and SDS with appropriate weight ratios were mixed and put into deionized water (30.0 mL). The mixture was probe ultrasonicated for 30.0 min to form a homogeneous suspended solution. The prepared suspended

solution was filtered through a membrane (pore size of 450 nm). After the filtered cake was dried, a MnO_2/CNTs electrode was obtained.

4.Preparation of acidic PAM hydrogel

Firstly, 3.0 g acrylamide, 30.0 mg $K_2S_2O_8$ and 4.0 mg N, N′-methylenebisacrylamide were sequentially added to 20.0 ml deionized water. Then the mixture was stirred for 1.0 h and injected into a glass model, followed by heating in an oven at 70 °C for 1.0 h. Finally, the as-fabricated hydrogel film was immersed in 2.0 M aqueous $(NH_4)_2SO_4$, 0.1 M $MnSO_4$ achieve the equilibrated state.

5. Materials characterization

The morphology and microstructure of the products were characterized using scanning electron microscopy (SEM, FEI Nova NanoSEM 450) and transmission electron microscopy (TEM, FEI Titan G2 60-300). The structure and phase purity of the as-synthesized products were revealed by X-ray diffraction (XRD, Rigaku X-ray diffractometer with Cu-Kα radiation). X-ray photoelectron spectrometry (XPS) analysis was carried out on an AXIS-ULTRA DLD-600W spectrometer. FTIR measurements were carried out on a Nicolet iS50R.

6. Electrochemical measurements

The electrochemical performances of electrodes, aqueous batteries and the AMBs including cyclic voltammetry (CV), galvanostatic charge and discharge curves (GCD) and electrochemical impedance spectroscopy (EIS) were tested using the

electrochemical workstation (AUT302N FRA2.V). The cycle life was performed on a LAND-CT2001A battery workstation.

Computational Details.

First-principles calculations based on density functional theory (DFT) are implemented in Quantum Espresso (QE) [J. Phys. Condens. Matter 21(39), 395502; J. Phys. Condens. Matter **29**(46), 465901]. The Perdew-Burke-Ernzerhof (PBE) functional of the generalized gradient approximation (GGA) is utilized to treat the exchange-correlation interaction [Phys. Rev. Lett. **77**(18), 3865]. A 6 × 6 × 1 supercell is used for Ti_3C_2OH monolayer with a vacuum slab of 15 Å. The convergence criteria for energy and force are set at 10^{-5} eV and 0.01 eV/Å, respectively. The cutoff energy of 500 eV is applied. The adsorption energies can be obtained by,

$$E_{\mathrm{ads}} = E_{\mathrm{Ti_3C_2OH\text{-}PTCDA}} - E_{\mathrm{Ti_3C_2OH}} - E_{\mathrm{PTCDA}}$$

where $E_{\mathrm{Ti_3C_2OH\text{-}PTCDA}}$, $E_{\mathrm{Ti_3C_2OH}}$ and E_{PTCDA} are the energies of Ti_3C_2OH with adsorbed PTCDA, the pure Ti_3C_2OH and the isolated PTCDA, respectively.

The specific capacity (Q) and specific capacitance (C) are respectively calculated from equations (1) and (2).

$$Q = \frac{I \cdot \Delta t}{m} \qquad (1)$$

$$C = \frac{I \cdot \Delta t}{m \cdot \Delta U} \qquad (2)$$

where I, Δt, m and ΔU represent the charge or discharge current, charge or discharge time, mass of active materials and potential window, respectively.

As the GCD curves of the devices are approximately linear, the corresponding energy density (E) and power density (P) are respectively calculated from equations (3) and (4).

$$E = 0.5 \cdot C \cdot \Delta U^2 \quad (3)$$

$$P = \frac{E}{\Delta t} \quad (4)$$

where the specific capacitance (C) can be obtained through equation (2), Δt is the discharge time.

The capacitance retention (CR) and coulomb efficiency (CE) are respectively calculated from equations (5) and (6).

$$CR = \frac{\Delta t}{\Delta t_0} \quad (5)$$

$$CE = \frac{\Delta t_d}{\Delta t_c} \quad (6)$$

where Δt is the discharge time of different cycles and Δt_0 is the initial discharge time, and Δt_d is the discharge time and Δt_c is the charge time in same cycle.

The battery was discharged or charged to make the voltage reach equilibrium. The D_{Zn} can be calculated based on the simplified equation of $D_{NH4+} = \frac{4}{\pi\tau}(\frac{m_B V_M}{M_B A})^2(\frac{\Delta E_S}{\Delta E_\tau})^2$, where m_B is the mass of the active material, M_B is the molecular weight, V_M is the molar volume, A is the total contact area between electrode and electrolyte, τ is the duration time of the current pulse, ΔE_τ is the variation of the battery voltage, and ΔE_S is related to the change of steady-state voltage for the corresponding step.

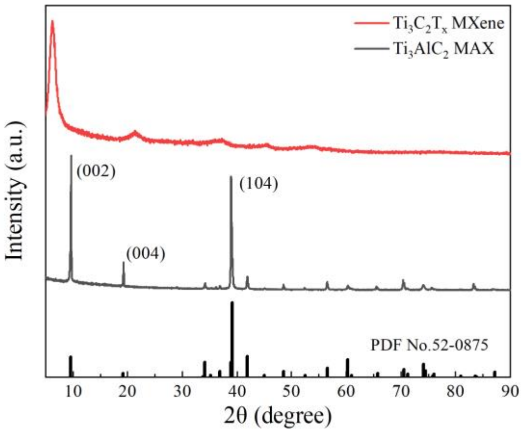

Figure S1. The XRD patterns of Ti_3AlC_2 MAX powder and $Ti_3C_2T_x$ MXene powder.

Figure S1 shows the XRD patterns of the MXene and MAX. After etching Al from MAX, the mainly typical peaks of MAX disappear and a new peak of around 7° appears, which means the successful preparation of MXene.

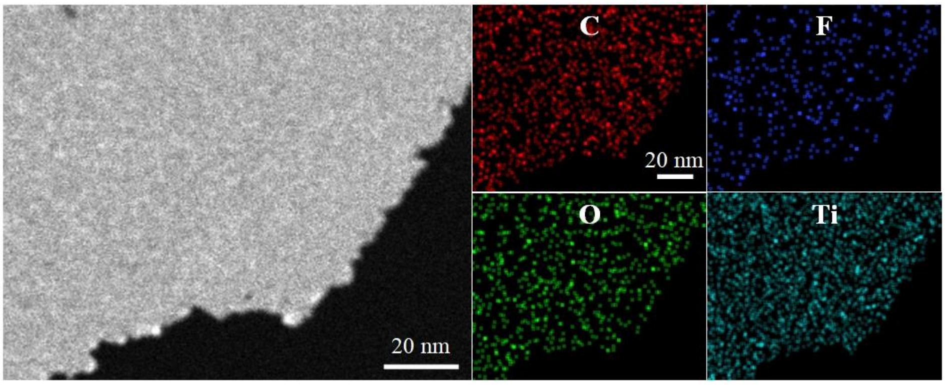

Figure S2. The TEM images, HAADF-TEM and STEM elemental mapping image of $Ti_3C_2T_x$ MXene powder.

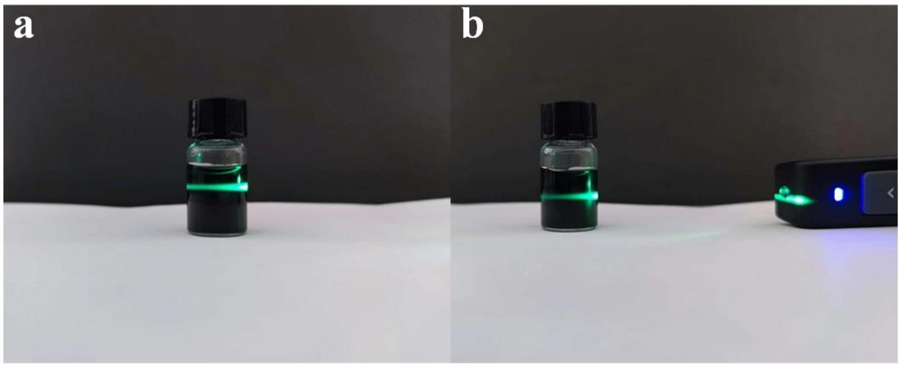

Figure S3. The Dendar effect of (a) aqueous $Ti_3C_2T_x$ MXene and (b) Organic phase $Ti_3C_2T_x$ MXene

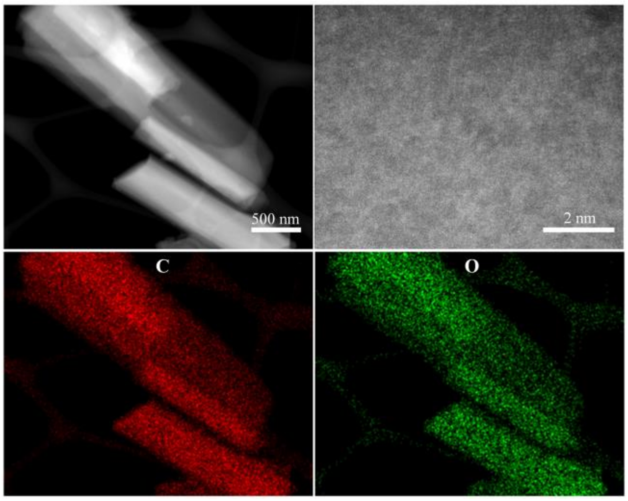

Figure S4. The TEM images of commercialized PTCDA powder.

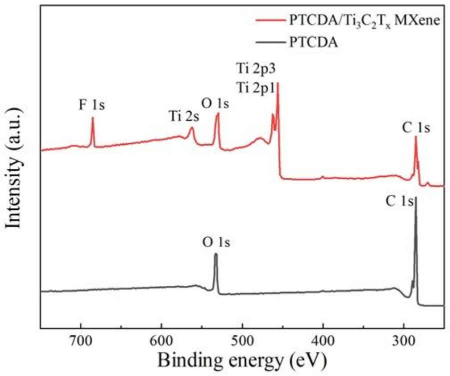

Figure S5. The XPS full spectrum of PTCDA powder and PTCDA/$Ti_3C_2T_x$ MXene free-standing film.

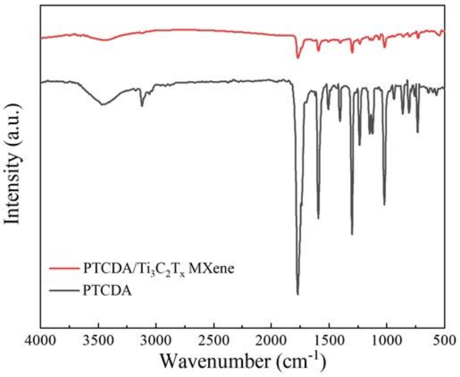

Figure S6. The FTIR spectrum of PTCDA powder and PTCDA/$Ti_3C_2T_x$ MXene free-standing film.

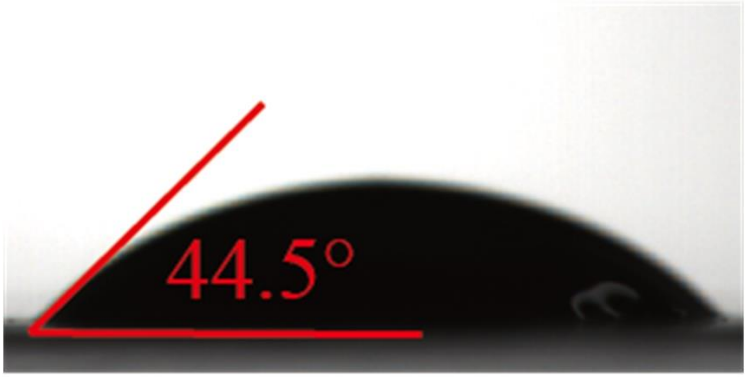

Figure S7. The contact Angle of PTCDA/$Ti_3C_2T_x$ MXene free-standing film and the electrolyte.

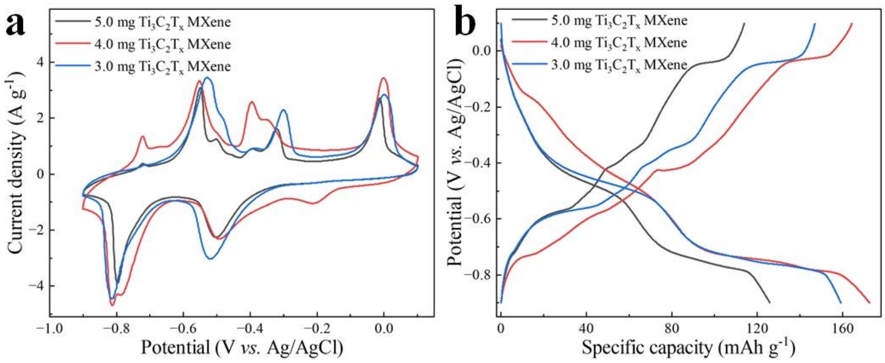

Figure S8. The CV curves at 2.0 mV s^{-1} and GCD curves at 1.0 A g^{-1} of PTCDA/$Ti_3C_2T_x$ MXene with different proportion.

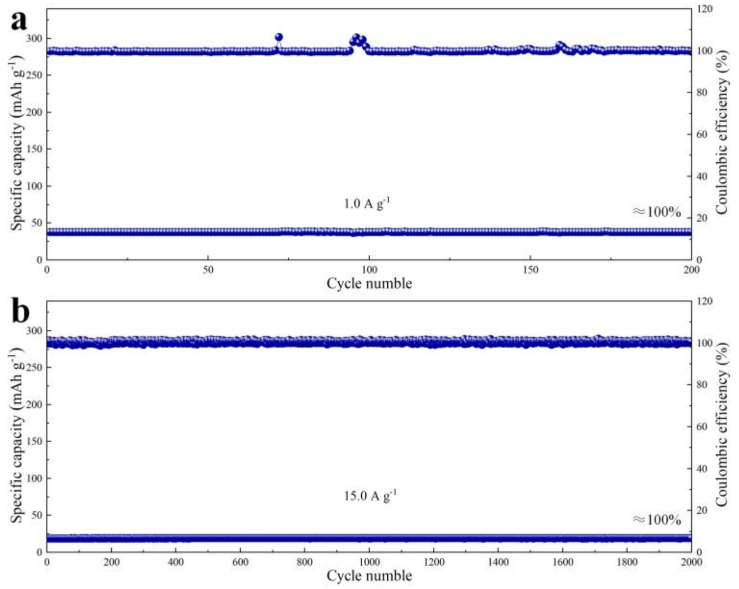

Figure S9. The cycle life at (a) 1.0 A g^{-1} and (b) 15.0 A g^{-1} of $Ti_3C_2T_x$ Mxene

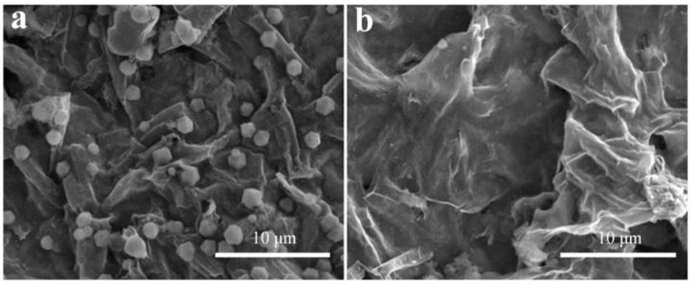

Figure S10. The SEM images of PTCDA/$Ti_3C_2T_x$ MXene after cycling at (a) 1.0 A g^{-1} and (b) 15.0 A g^{-1}.

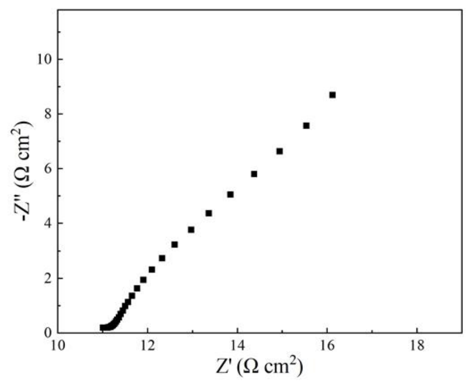

Figure S11. The EIS curves of PTCDA/$Ti_3C_2T_x$ MXene.

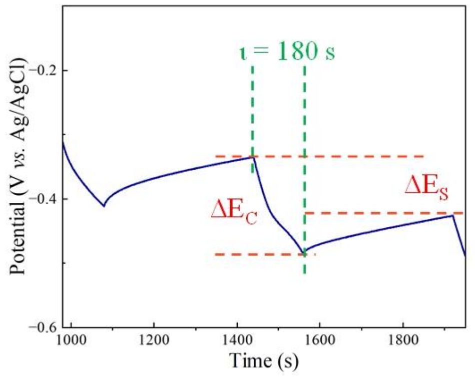

Figure S12. The schematic illustration of selected steps of the GITT curve.

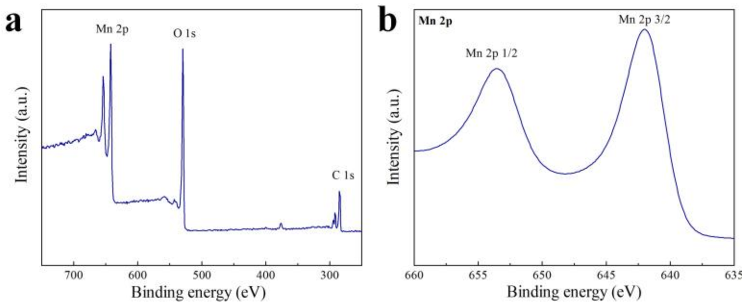

Figure S13. The a) full XPS spectra and (b) Mn 2p XPS spectrum of the MnO_2.

From Mn 2p XPS spectra, the peak of 642.6 eV (Mn 2p3) and 654.3 eV (Mn 2p1/2) with a spin-energy separation of 11.7 eV can be observed.

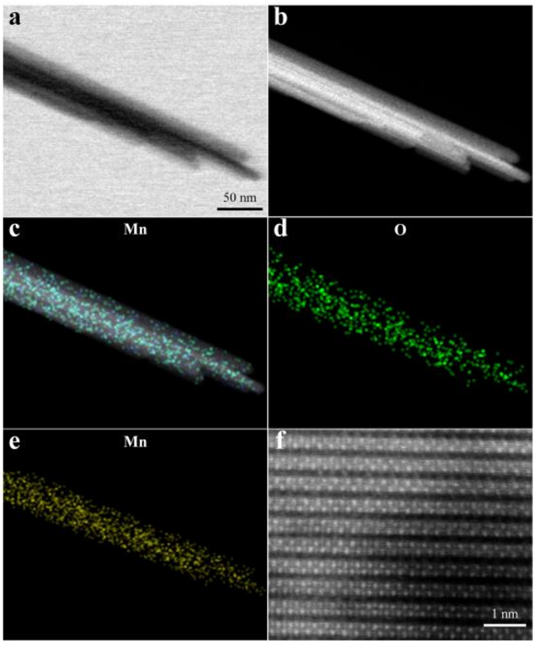

Figure S14. The TEM images, TEMHAADF-TEM and STEM elemental mapping image of MnO_2. The TEM images demonstrated the nanowire structure of MnO_2.

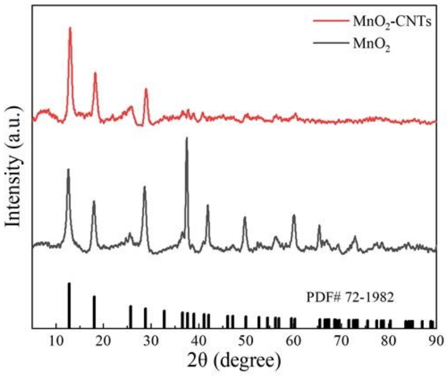

Figure S15. The XRD patterns of the MnO_2 and the MnO_2-CNTs.The peaks of MnO_2-CNTs consists of the peaks of MnO_2 and CNTs in XRD patterns, indicating the successful synthesis of MnO_2-CNTs.

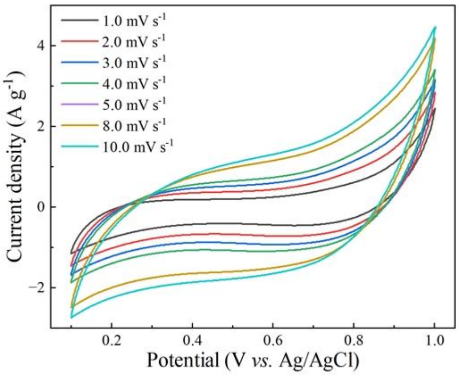

Figure S16. The CV curves from 1.0 m V s^{-1} to 10.0 m V s^{-1} of MnO_2/CNTs.

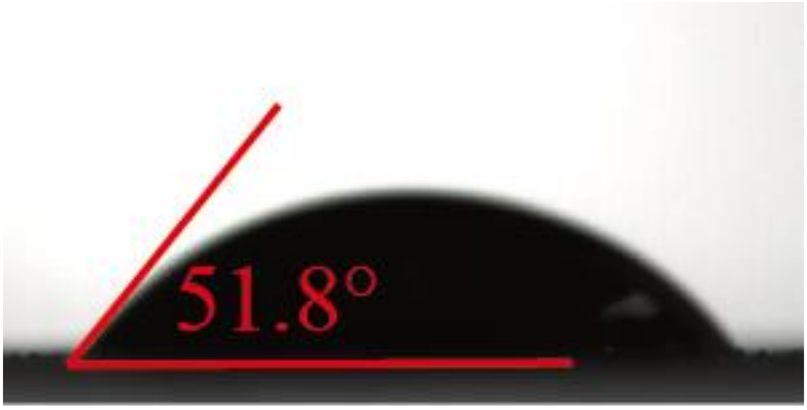

Figure S17. The contact Angle of MnO_2/CNTs free-standing film and the electrolyte.

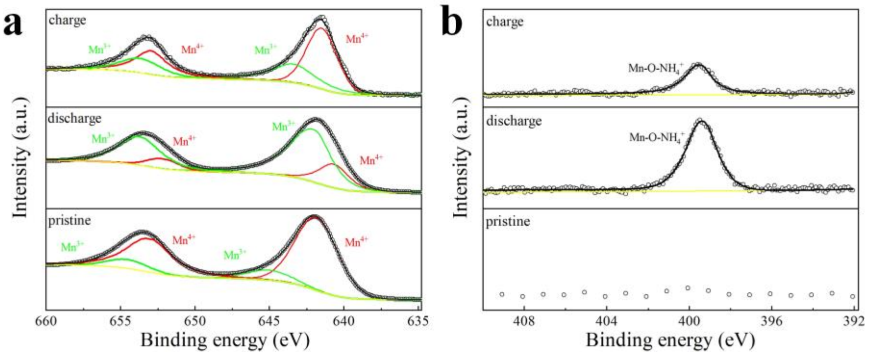

Figure S18. The XPS spectrum of MnO_2/CNTs electrode.

As shown in Figure s18a, during the discharge process, the Mn^{4+} decreases and the Mn^{3+} appears, indicating that NH_4^+ insertion into MnO_2. During the discharge, the recovery of Mn^{4+} and the decrease of Mn^{3+} indicate that the insertion/extraction of NH_4^+ with MnO_2/CNTs electrode is reversible. It is worth noting that the appearance and disappearance of Mn-O-NH_4^+ during discharge/charging is also important evidence of reversible NH_4^+ insertion/extraction (Figure s18b).

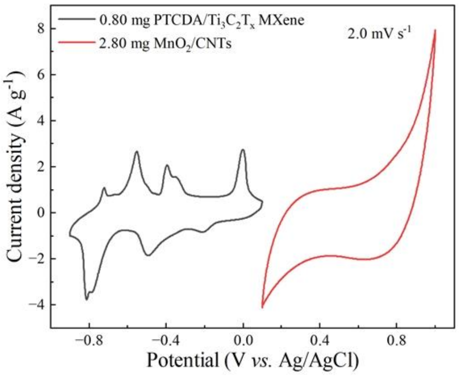

Figure S19. The CV curves at 2.0 mV s^{-1} of PTCDA/$Ti_3C_2T_x$ MXene anode and MnO_2/CNTs cathode.

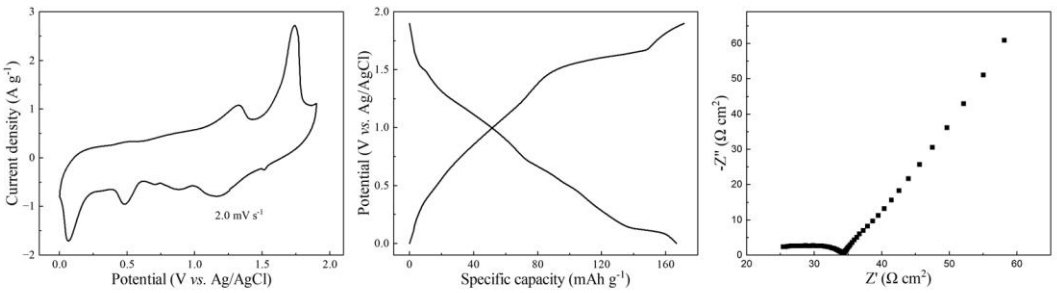

Figure S20. The performance of aqueous batteries. (a) CV curves at 2.0 mV s^{-1}, (b) GCD curves at 1.0 A g^{-1} (based anode mass) and (c) EIS curves.
